# Influence of Posttraumatic Stress Disorder Severity on Return to Substance Use Immediately Following Residential Substance Use Treatment

**DOI:** 10.1007/s11469-024-01374-1

**Published:** 2024-09-16

**Authors:** Nicole H. Weiss, Noam G. Newberger, Emmanuel D. Thomas, Silvi C. Goldstein, Diana Ho, Stephen M. Coutu, Alyssa L. Avila, Ateka A. Contractor, Lynda A. R. Stein

**Affiliations:** 1 Department of Psychology, University of Rhode Island, 142 Flagg Rd., Kingston, RI 02881 USA; 2 Brown University, Providence, RI USA; 3 Department of Psychology, University of North Texas, Denton, TX USA; 4 Department of Behavioral Healthcare, Developmental Disabilities, & Hospitals, Cranston, RI USA

**Keywords:** Posttraumatic stress disorder, Substance use disorder, Return to substance use, Residential substance use treatment

## Abstract

The period immediately following residential substance use disorder (SUD) treatment is characterized by high rates of return to substance use. Posttraumatic stress disorder (PTSD) is highly prevalent among individuals in residential SUD treatment and is a primary motive for substance use among individuals with co-occurring PTSD and SUD. Addressing important gaps in the literature, the current study examined the role of PTSD severity on days of substance use during the 30 days immediately following residential SUD treatment over and above demographic, SUD, and clinical factors associated with return to substance use. Participants (*N* = 65, M_age_ = 40.6, 52% women, 79% white) completed semi-structured diagnostic interviews for PTSD and SUD and self-report measures of demographics and depression while in residential SUD treatment (approximately one week before discharge), and then a follow-up assessment (timeline follow-back for substance use) approximately one month after discharge. Greater PTSD severity was associated with more days of substance use in the 30 days immediately following residential SUD treatment over and above demographic (i.e., race/ethnicity, gender, employment, housing insecurity), SUD (i.e., alcohol, stimulant, opioid, cannabis, and sedative/hypnotic/anxiolytic use disorder severity), and clinical (i.e., depression severity) factors. Findings underscore the importance of PTSD assessment and intervention during residential SUD treatment and re-entry planning to assist in mitigating return to substance use during community reintegration.

Substance use disorder (SUD) is a major public health crisis. In 2021, 46.3 million people in the United States aged 12 years or older (or 16.5% of the population) had an SUD in the past year, including 29.5 million who had an alcohol use disorder and 24.0 million who had a drug use disorder (Substance Abuse & Mental Health Services Administration [SAMSHA], 2022). SUD incurs a staggering societal cost and contributes heavily to disease burden. At the individual level, SUD is linked to substantial impairment in major life roles, diminished quality of life, deleterious health outcomes (e.g., HIV/AIDS), increased risk for suicidality, and neuropsychological deficits (Degenhardt & Hall, 2012; Swendsen & Merikangas, 2000; Yuodelis-Flores & Ries, 2015). SUD also confers considerable burden on families, social networks, and society as a whole via increases in health care usage, crime, poverty, homelessness, and productivity loss (Bouchery et al., 2011; Degenhardt & Hall, 2012). The United States has seen increasing rates of these SUD-related harms over the past two decades (White, 2020). Altogether, it is estimated that the yearly economic impact of substance use is $249 billion for alcohol use and $193 billion for drug use (Murthy, 2017). These figures underscore SUD’s high prevalence and significant impact.

Effective SUD treatment is a clinical and public health priority. Community reintegration following residential SUD treatment represents a critical period. Over 13 million people in the United States aged 12 years or older sought treatment for SUD in the past year; this translates to 4.6% of people in the United States aged 12 years or older and 24% of people in the United States aged 12 years or older who needed substance use treatment in the past year (SAMSHA, 2022). Roughly 3.5 million people in the United States aged 12 years or older received inpatient (e.g., residential) SUD treatment. Residential SUD treatment is a structured, 24-h level of care that enables a focus on intensive recovery activities. It aims to help people with SUD achieve stability in a safe setting before returning to an unsupervised environment, which may otherwise be detrimental to their recovery goals. Residential SUD treatment has shown promise in reducing substance use as well as related health and social costs (Reif et al., 2014). Yet, SUD is characterized by high rates of return to substance use following treatment (40–70%), with most individuals with SUD cycling through periods of substance use, treatment re-entry, and recovery (Scott et al., 2005). Risk for return to substance use is greatest during the first three months of recovery (Sinha, 2011). Thus, it is critical to identify factors associated with return to substance use following residential SUD treatment to prevent and/or delay return to substance use during this high-risk transitional period.

Posttraumatic stress disorder (PTSD) is an important factor to consider in this regard. Nearly all patients with an SUD (97.4%) report a history of trauma (Gielen et al., 2012). PTSD is etiologically tied to trauma and characterized by intrusions, avoidance of trauma cues, negative cognitions and mood, and arousal and reactivity (American Psychiatric Association, 2013). Roughly 8% of individuals in the general population develop PTSD in their lifetime (Kilpatrick et al., 2013). However, among patients with SUD, rates of current and lifetime PTSD rise disproportionately to 25–42% and 36–50%, respectively (Jacobsen et al., 2001). The high co-occurrence of PTSD and SUD is an urgent health priority. Compared to individuals with either PTSD or SUD alone, those with co-occurring PTSD and SUD have more chronic and severe patterns of substance use, greater physical and psychiatric comorbidity, and increased functional impairment (Brady et al., 2021). Moreover, the presence of PTSD among patients with SUD is associated with a more complicated course of treatment and less favorable treatment outcomes compared that of PTSD or SUD alone (Flanagan et al., 2016; Tripp et al., 2019).

However, a dearth of research has examined the impact of PTSD on return to substance use (Bradizza et al., 2006). Brown et al. (1996) assessed return to use among 31 women during the three months following inpatient substance use treatment at a private psychiatric hospital. Most of the women (70%) returned to use during this three-month period. Although the rate of return to substance use did not significantly differ as a function of PTSD status, women with co-occurring PTSD and SUD returned to substance use significantly more quickly than women with SUD alone. Similarly, among 1,480 male Veterans who received inpatient treatment for substance use, Ouimette et al. (1999) found that those with co-occurring PTSD and SUD (versus those with SUD and another psychiatric disorder and those with SUD alone) were less likely to be in remission (i.e., abstinent from alcohol and drug use or meeting the following criteria: consumption of 3 oz or less of ethanol on a usual drinking day, no problems resulting from alcohol and drug use, and no illicit drug use) at 1- and 2-year follow-ups. Further, those with co-occurring PTSD and SUD consumed significantly more alcohol and reported significantly more substance-related harms at 1- and 2-year follow-ups compared to those with SUD and another co-occurring psychiatric disorder and those with SUD alone. The limited research in this area represents a critical gap in the literature given evidence that a primary motive for substance use among individuals with co-occurring PTSD and SUD is relief from PTSD symptoms (Leeies et al., 2010; Luciano et al., 2022). To our knowledge, no study has examined the role of PTSD in return to substance use immediately after residential SUD treatment in the general population.

Addressing this critical gap in the literature, the current study examined the role of PTSD on return to substance use during the 30 days following residential SUD treatment in a community sample. To assess the unique role of PTSD, other established correlates of return to substance use were included in the model, including demographic (i.e., gender, race/ethnicity, employment, and housing), SUD (i.e., alcohol and other drug severities), and clinical (i.e., depression) factors. Sliedrecht et al. (2019) highlights several of these factors in their meta-analytic review: women and individuals who were employed were less likely to return to substance use, whereas individuals with psychiatric comorbidity (inclusive of depression) and more severe SUD were more likely to return to substance use. Other research underscores the influence of racial and ethnic background on return to substance use, with evidence to suggest that the negative effects of social and structural determinants of recovery are compounded among individuals with a systemically marginalized racial and/or ethnic background (Mumba et al., 2023). Housing instability is another factor that has been found to be relevant to return to substance use, with evidence that substance use is characterized by a chronic relapsing course among individuals who experience housing insecurity (Scott et al., 2005). We hypothesized that greater PTSD severity (assessed in residential SUD treatment, within one week of discharge) would be associated with more days of substance use following residential SUD treatment over and above these demographic, SUD, and clinical factors.

## Methods

### Participants

Participants were recruited from two residential SUD treatment facilities in the northeast. The typical length of treatment in these facilitates was one month, although this duration varies quite substantially, from a few weeks to a few months. Research assistants approached individuals who were scheduled to discharge within one week. If interested, potential participants were brought to a private room, where eligibility was determined through administration of screening questions (self-report). Inclusion criteria were: (1) aged 18 years or older, (2) fluent in the English language, (3) owning a smartphone, (4) history of traumatic experiences, and (5) scheduled to discharge from the residential SUD treatment facility in 7 days or less. Exclusion criteria were (a) presence of current mania or psychosis (assessed in the baseline session with the Structured Clinical Interview for the Diagnostic and Statistical Manual of Mental Disorders, Fifth Edition [DSM-V; SCID-5]; First & Williams, 2016) and (b) current impairment in cognitive functioning (assessed in the baseline session using the Mini-Mental Status Exam and requiring a score > 24; Folstein et al., 1975).

### Procedures

All procedures were reviewed and approved by the University of Rhode Island Institutional Review Board. At the end of each session, participants were given a list of community resources. Assistance with referrals was provided upon participant request. The principal investigator (author NHW), a licensed clinical psychologist in the state of Rhode Island, was available on-call if participants required additional trauma- and/or substance-related support.

#### Baseline Session

Baseline sessions were conducted by a bachelors- or masters-level clinical psychology doctoral student in a private office in the residential SUD treatment facility to protect participants’ safety and confidentiality. The baseline session was conducted within one week of a participant’s scheduled discharge. After providing informed consent, participants were interviewed using structured diagnostic assessments and then answered self-report measures. Participants were compensated with $25 for completing the baseline session.

#### Follow-up Session

Follow-up sessions occurred approximately 32–35 days after discharge from residential SUD treatment. They were completed by a study research assistant in a private research office to protect participants’ safety and confidentiality. Participants were interviewed using a timeline follow-back (TLFB) method and answered self-report measures on a computer. Participants were compensated with $40 for completing the follow-up session.

### Measures

#### Diagnostic Measures

Diagnostic interviews were administered during the baseline session by clinical psychology doctoral students who were trained to reliability with the principal investigator. All diagnoses were reviewed and confirmed by the principal investigator in consensus meetings.

##### PTSD Severity

The Clinician-Administered PTSD Scale for DSM-5 (CAPS-5) (Weathers et al., 2013) is a semi-structured interview for PTSD that uses the DSM-5 (American Psychiatric Association, 2013) diagnostic criteria to establish a past-30-day continuous PTSD severity score (i.e., CAPS-5 total score) and diagnostic label (i.e., present vs. absent) for each participant. The CAPS-5 first assesses the presence of a Criterion A trauma. For participants with a history of Criterion A trauma, severity ratings (that consider both frequency and intensity of experiences) are given for the 20 DSM-5 PTSD symptoms. Severity items are rated from 0 (absent) to 4 (extreme/incapacitating). Severity ratings for the 20 DSM-5 PTSD symptoms are then summed to create a total PTSD severity score. PTSD severity ranges from 0 to 80, with higher scores indicating greater severity of PTSD. After rating the severity of DSM-5 PTSD symptoms, criterion F (duration of disturbance ≥ 1 month) and G (subjective distress and/or impairment in functioning) are assessed, global ratings for validity and severity are indicated, and two dissociative symptoms are evaluated for the dissociative subtype of PTSD. The CAPS-5 has demonstrated excellent psychometrics (Weathers et al., 2018). Internal consistency for the total PTSD severity score in the current sample was excellent (*α* = 0.91).

##### SUD Severity

The Structured Clinical Interview for DSM-5 (SCID-5) (First & Williams, 2016) is a semi-structured interview for DSM-5 diagnoses (American Psychiatric Association, 2013). In the current study, the SCID-5 was administered to establish current and lifetime alcohol and drug (i.e., sedative/hypnotic/anxiolytic, cannabis, stimulant, opioid, inhalant, PCP, hallucinogen, other/unknown) use disorders. The SCID-5 SUD modules first assess lifetime and current (past 30-day) history of alcohol and drug use. For participants with a history of alcohol and/or drug use, severity ratings are provided for each of the 11 DSM-5 SUD symptoms for each substance that is endorsed; this is done for both lifetime (prior to past 12 month) and current (past 12 month) disorders. Severity ratings were absent (1), subthreshold (2), and present (3). These were recoded to absent (0), subthreshold (0), and present (1) and then summed, with higher scores indicating the presence of more alcohol/drug use symptoms. As such, potential SUD severity scores could range from absent (0) to most severe (11). The SCID-5 has demonstrated moderate to excellent inter-rater reliability, including sensitivity of 0.90, specificity of 0.99, and a κ of 0.92 for any SUD (Osório et al., 2019). Internal consistency in the current sample was good for the alcohol and drug use disorders (αs ranging from 0.77 to 0.78).

#### Self-report Measures

##### Demographics

All participants completed a demographics form during the baseline session that included items assessing age, gender, race, ethnicity, employment, and housing insecurity. Housing insecurity was measured with two dichotomous (no = 0, yes = 1) items which were summed, with higher scores indicative of more housing insecurity. Given the small number of participants in several of the gender, race, ethnicity, and employment categories, these variables were collapsed into dichotomous variables of men (0) versus women (1); non-Hispanic white (0) versus member of a systemically marginalized racial and/or ethnic group (1); and not employed (0) versus some employment (1).

##### Depression

The Patient Health Questionnaire – 9 (PHQ-9) (Kroenke & Spitzer, 2002) is a 9-item self-report measure that assesses depression symptoms over the past two weeks. Participants completed the PHQ-9 during the baseline session. The four response options range from 0 (not at all) to 3 (nearly every day). Higher scores indicate greater depression severity. The PHQ-9 has demonstrated good reliability and validity (Kroenke et al., 2001). Internal consistency in the current sample was excellent (*α* = 0.89).

##### Return to Substance Use

The TLFB (Sobell & Sobell, 1992) assessed the presence of alcohol and drug use for the 30 days immediately after leaving residential SUD treatment. The day of discharge was assigned as Day 1. A calendar format was used to provide temporal cues to assist in recall. Types of drugs assessed included sedatives/hypnotics/anxiolytics, cannabis, stimulants/amphetamines, cocaine, hallucinogens, PCP, MDMA, inhalants, and other. Interviewers distinguished between medication assisted treatment (MAT) and non-medical prescription use. Specifically, when participants endorsed prescription medication use, they were asked whether the prescription medication(s) were not prescribed to them or whether used the prescription drug(s) not as prescribed (e.g., took more than prescribed or took for a longer period of time than prescribed). Dichotomous scores were created indicating any alcohol or drug use (yes [1]/no [0]) for each of the 30 days. These scores were summed to reflect number of days of substance (alcohol or drug) use (range 0 to 30). A score of ≥ 1 indicated that the participant returned to substance use. The TLFB exhibits good reliability (Sobell et al., 1996) and concurrent validity with other substance use measures (DeMarce et al., 2007).

### Data Management and Analytic Strategy

Our dependent variable was number of days of substance (alcohol or drug) use during the 30 days following residential SUD treatment. The final model included demographic (i.e., race/ethnicity, gender, housing insecurity, employment), SUD (i.e., alcohol, stimulant, opioid, cannabis, and sedative/hypnotic/anxiolytic use disorder severity), and clinical (depression and PTSD severity) covariates. Inhalant (*n* = 1), PCP (*n* = 1), and hallucinogen (*n* = 3) use disorder severity were excluded from the model due to low endorsement. Depression severity scores were imputed for four participants who were each missing a single item from the PHQ-9. We initially tested a Poisson model, but this model was overdispersed. The dependent variable was not zero-inflated so a negative binomial regression model with a maximum likelihood bias reduction was used to assess relations between demographic, SUD, and clinical covariates and return to substance use. We also tested a second, adjusted model to account for the sample size of the current study and possible overfitting of the final model. This adjusted model excluded SUD severity variables and only included demographic variables and clinical correlates (i.e., depression and PTSD severity). Both models used a log link and identity transformation of the dispersion parameter. Missing data was omitted listwise. We tested the models using the brnb function in the brglm2 package (Kosmidis et al., 2023). Model results are reported as incidence rate ratios (IRR) with 95% confidence intervals.

## Results

### Descriptive Results

The final sample included 65 individuals who participated in both the baseline and follow-up sessions. Participants ranged in age from 19 to 63 years old (*M* = 42.41, *SD* = 10.67). Forty-three (66.2%) of the participants identified as women and 21 (32.3%) identified as men; one participant (1.5%) declined to identify their gender. Most of the participants identified their race to be white (*n* = 47, 72.3%), with seven (10.8%) identifying as Bi- or Multi-racial, six (9.2%) as Black or African American, and one (1.5%) as Native Hawaiian or Pacific Islander. Four other participants indicated that their racial background was not listed: two (3.1%) identified their race as Cape Verdean, one (1.5%) as Middle Eastern, and one (1.5%) as Puerto Rican. The majority of the participants identified their ethnicity to be non-Hispanic or Latino/a (*n* = 60, 92.3%); four (6.2%) identified as Hispanic or Latino/a and one (1.5%) declined to identify their ethnicity. Fifty-one (78.5%) of the participants reported that they were unemployed, four (6.2%) were not in the labor force (e.g., retired, student), six (9.2%) were employed part-time, and three (4.6%) were employed full-time; one (1.5%) declined to report their employment status. Twenty-one (32.3%) of the participants reported less than a high school degree, 17 (26.2%) reported a high school degree or equivalent, and 27 (41.5%) reported some college education. Approximately one-third of the participants reported an annual income less than $9,999 (*n* = 22, 33.8%); five (7.7%) reported an annual income between $10,000 and $19,999; 12 (18.5%) an annual income between $20,000 and $29,999; 14 (21.5%) an annual income between $30,000 and $39,999; and 11 (16.9%) an annual income greater than $40,000; one (1.5%) declined to report their income. Finally, 26 (40.0%) of the participants reported that they were not dating, 23 (35.4%) that they were dating, three (4.6%) that they were married, and 11 (16.9%) that they were separated or divorced; two (3.1%) declined to report their relationship status. Demographic variables for the current study are presented in Table 1.

**Table 1:** Sample demographic characteristics

	*M* (*SD*)	Range	*n* (%)
Age	42.41 (10.67)	19 – 63	
Gender			
Women			43 (66.2%)
Men			21 (32.3%)
Prefer not to respond			1 (1.5%)
Racial/Ethnic background			
Black or African American			6 (9.2%)
Native Hawaiian/Pacific Islander			1 (1.5%)
Bi-/multi-racial			7 (10.8%)
White			47 (72.3%)
Not listed^a^			4 (6.2%)
Ethnicity			
Hispanic or Latinx			4 (6.2%)
Non-Hispanic or Latinx			60 (92.3%)
Prefer not to respond			1 (1.5%)
Highest education completed			
Grades 6–8			3 (4.6%)
Grades 9–11			18 (27.7%)
High School			17 (26.2%)
College/Professional school (1–4 years)			23 (35.4%)
College/Professional school (5 + years)			4 (6.2%)
Employment			
Full time (35 + hours per week)			3 (4.6%)
Part time (< 35 h per week)			6 (9.2%)
Unemployed			51 (78.5%)
Not in labor force			4 (6.2%)
Prefer not to respond			1 (1.5%)
Monthly household income			
$0-$9,999			22 (33.8%)
$10,000-$19,999			5 (7.7%)
$20,000-$29,999			12 (18.5%)
$30,000-$39,999			14 (21.5%)
$40,000 +			11 (16.9%)
Prefer not to respond			1 (1.5%)
Relationship status			
Not dating			26 (40%)
Dating			23 (35.4%)
Married			3 (4.6%)
Separated or divorced			11 (16.9%)
Prefer not to respond			2 (3.1%)

^a^Of the participants who indicated that their racial background was not listed, two self-described as Cape Verdean, one as Middle Eastern, and one identified as Puerto Rican

Over one-third of participants (35.4%) reported substance use in the 30 days immediately following residential SUD treatment. In the 30 days immediately following residential SUD treatment, participants reported an average of 10.83 days of substance use (*SD* = 10.06, *Median* = 6, *range* = 1 – 30), and, on average, returned to substance use after 9.65 days following discharge (*SD* = 8.45, *Median* = 10, *range* = 1 – 30). Among those that returned to any substance use, 16.9% returned to alcohol use. These individuals reported an average of 7.55 days of alcohol use (*SD* = 6.12, *Median* = 4, *range* = 1 – 18) in the 30 days immediately following residential SUD treatment, and, on average, returned to alcohol use after 10.82 days following discharge (*SD* = 9.02, *Median* = 12, *range* = 1 – 30). Among those that returned to substance use, 23.1% returned to drug use. These individuals reported an average of 11.93 days of drug use (*SD* = 11.16, *Median* = 7, *range* = 1 – 30) in the 30 days immediately following residential SUD treatment, and, on average, returned to drug use after 8.2 days following discharge (*SD* = 7.46, *Median* = 5, *range* = 1 – 26).

### Primary Results

See Table 2 for findings from the regression analysis. Consistent with our hypothesis, PTSD severity was significantly positively associated with days of substance use during the 30 days immediately following residential SUD treatment. Specifically, there was a 5% increase in the incidence rate of days of substance use per one-unit increase in PTSD severity score (*IRR* = 1.05, 95% CI [1.00, 1.11], *p* = 0.033). Race/ethnicity, housing, and employment were also significantly associated with days of substance use. The incidence rate of days of substance use for participants who were members of systemically marginalized racial and/or ethnic groups was 87% lower for participants compared to the non-Hispanic white group (IRR = 0.13, 95% CI [0.02, 0.70], *p* = 0.017). A one-unit decrease in housing insecurity score was associated with a 69% reduction in the rate of days of substance use (IRR = 0.32, 95% CI [0.10, 0.97], *p* = 0.044). The incidence rate of days of substance use for participants who reported some employment was 97% lower compared to participants who were unemployed (IRR = 0.03, 95% CI [0.00, 0.30], *p* = 0.003).

**Table 2:** Rates of days of substance use

Variables	Incidence rate ratios	95% confidence intervals	*p*
Adjusted model			
Systemically marginalized racial and/or ethnic group	0.34	0.08 – 1.56	0.166
Women	0.18	0.04 – 0.77	0.021
Housing insecurity	0.51	0.18 – 1.41	0.194
Employment	0.08	0.01 – 0.65	0.019
Depression severity score	0.88	0.78 – 1.00	0.042
PTSD severity score	1.05	1.01 – 1.10	0.026
Final model			
Systemically marginalized racial and/or ethnic group	0.13	0.02 – 0.70	0.017
Women	0.22	0.05 – 1.02	0.053
Housing insecurity	0.31	0.10 – 0.97	0.044
Employment	0.03	0.00 – 0.30	0.003
Depression severity score	0.93	0.82 – 1.05	0.238
PTSD severity score	1.05	1.00 – 1.11	0.033
AUD severity score	1.39	0.86 – 2.26	0.181
SAH severity score	1.51	0.80 – 2.83	0.204
CUD severity score	1.09	0.56 – 2.13	0.800
OUD severity score	0.57	0.31 – 1.04	0.068
SUD severity score	1.54	0.92 – 2.57	0.102

*AUD* Alcohol use disorder; *SAH* Sedative/Anxiolytic/Hypnotic use disorder; *CUD* Cannabis use disorder; *OUD* Opioid use disorder; *SUD* Stimulant use disorder; *PTSD* Posttraumatic stress disorder. Reference categories for categorical covariates are (in order) Non-Hispanic white, Men, and Unemployed

Given the smaller sample size, we also assessed an adjusted model that excluded all SUD severity variables and included only demographic variables (i.e., marginalized racial and/or ethnic group, gender, housing insecurity, employment) and clinical characteristics (i.e., depression and PTSD severity; see Table 2). In this adjusted model, gender (IRR = 0.18, 95% CI [0.04, 0.77], *p* = 0.021), employment (IRR = 0.08, 95% CI [0.01, 0.65], *p* = 0.019), depression severity (IRR = 0.88, 95% CI [0.78, 1.00], *p* = 0.042), and PTSD severity (IRR = 1.05, 95% CI [1.01, 1.10],* p* = 0.026) were significantly associated with the incidence rate of days of substance use.

## Discussion

The goal of the current study was to examine the role of PTSD severity on return to substance use during the 30 days immediately following residential SUD treatment, over and above demographic, SUD, and clinical factors associated with return to substance use. Over one-third of participants returned to substance (alcohol and/or drug) use during the 30 days after residential SUD treatment. Among participants who returned to substance use, return to substance use occurred, on average, after 10 days, and substance use was reported on 11 (of the 30) days, on average. These preliminary findings underscore immediate community reintegration following residential SUD treatment as a high-risk period for return to substance use.

Consistent with our hypothesis, higher PTSD severity was associated with a greater number of days of substance use during the 30 days following residential SUD treatment. These findings align with theoretical explanations, empirical evidence, and treatment studies for co-occurring PTSD and SUD. The self-medication model suggests that return to substance use may function to escape or avoid PTSD symptoms (Khantzian, 1997). Empirical evidence provides robust support for the self-medication model in co-occurring PTSD and SUD (for a systematic review, see Hawn et al., 2020), suggesting a central function of down-regulation of trauma-related distress in (return to) substance use among individuals with PTSD. Indeed, relief from PTSD symptoms has been found to be a primary motive for substance use among individuals with co-occurring PTSD and SUD (Leeies et al., 2010; Luciano et al., 2022). Perhaps not surprising then, persistence of PTSD symptoms after substance use treatment has been shown to predict worse substance use outcomes, as individuals with PTSD continue to experience symptoms that have served as a prominent precipitant of substance use (Bradizza et al., 2006). Conversely, Ouimette et al. (2003) found that patients with SUD who received outpatient PTSD treatment shortly after discharge from substance use treatment (i.e., during the first 3 months) were more likely to be in SUD remission 5 years later. Collectively, these findings suggest that PTSD may play a critical role in return to substance use during community reintegration and that PTSD interventions provided during this time may enhance sustained remission for individuals with co-occurring PTSD and SUD.

In conjunction with existing literature, our findings underscore the need for interventions that address co-occurring PTSD and SUD for individuals in residential SUD treatment. Early approaches to the treatment of co-occurring PTSD and SUD followed the sequential model, which required patients to establish and maintain abstinence from substance use before initiating PTSD treatment (Flanagan et al., 2016). Use of the sequential model stemmed from providers’ concerns that PTSD treatment would lead to a worsening of SUD in those with PTSD (Back et al., 2009). Over time, however, these concerns have been dispelled (Lancaster et al., 2020), and the benefits of treating PTSD and SUD concurrently has become evident. Notably, however, while PTSD treatment has been demonstrated to be safe, acceptable, and efficacious for those with co-occurring SUD (Meshberg-Cohen et al., 2021), and integrated treatments for co-occurring PTSD and SUD have been shown to be more effective than treatment of individual disorders separately or in sequence (van Dam et al., 2012), SUD programs rarely assess trauma or provide PTSD treatment (Hien et al., 2000). The need for addressing this gap in translating research to practice is further magnified by evidence that patients prefer integrated treatment for PTSD and SUD (Back et al., 2006). Future research is needed to examine the benefits of providing integrated treatment for PTSD and SUD during—or immediately following—residential SUD treatment to prevent and/or delay return to substance use.

It also warrants discussion that several demographic factors were associated with number of days of substance use during the period of community reintegration. Individuals who were from systemically marginalized racial and/or ethnic groups, were employed, and were experiencing housing insecurity reported fewer days of substance use. These findings are partially consistent with extant research. For instance, while individuals from systemically marginalized racial and/or ethnic groups experience disproportionate levels of substance-related harm (Mulia et al., 2009; Zapolski et al., 2014), there is a large body of literature that provides evidence for lower rates of substance use among individuals from systemically marginalized racial and/or ethnic groups compared to their white counterparts (SAMSHA, 2022). Further, employment has generally been shown to be associated with long-term positive outcomes following SUD treatment (Henkel, 2011). Lastly, while research suggests that the stressors associated with housing insecurity may increase risk for return to use (Austin et al., 2021), it may have been the case that participants in this study who experienced housing insecurity were in a shelter or group home that prohibited substance use during community reintegration, which may have served as a strong motivator to abstain from substances or reduce substance use. Future research is needed to explore these hypotheses in larger samples over longer periods of time.

Notably, some of the demographic and clinical covariates associated with number of days of substance use during community reintegration differed between the primary model and an adjusted model that excluded SUD severity variables. Specifically, in the adjusted model, membership in a systemically marginalized racial and/or ethnic group and housing insecurity were no longer significantly associated with number of days of substance use, and gender and depression severity were now significantly associated with number of days of substance use. These mixed findings underscore the need for larger investigations that examine covariates of substance use during community reintegration. For instance, given divergent findings between models with and without SUD severity variables, there is a need to examine interactions between SUD and demographic and clinical covariates in relation to substance use during community reintegration.

While the current study has a number of strengths, including examination of a severely understudied population, use of diagnostic assessments and TLFB methods, and evaluation of relations over time (and during a high-risk transition period), several limitations warrant consideration. First, as the goal of the larger study was to establish research procedures for studying return to substance use in this population, the sample size was sufficient for the analytic approach but not large. Relatedly, the current study was not powered to examine return to alcohol and drug use separately, or return to specific classes of drug use (e.g., opioids, stimulants). Larger investigations with this population that address these questions are underway. Second, the outcome of focus was substance use. While total abstinence from substances may be a primary recovery goal and long-term treatment preference for some individuals exiting residential SUD treatment, others may prefer approaches that reduce use, minimize harms, and improve health and quality of life without requiring abstinence (Hay et al., 2019). Harm reduction approaches meet people “where they’re at” with compassion and respect (Collins et al., 2011, 2019, 2021), and prioritize client-centered goals, allowing for more flexible and attainable goals than standard strategies with a predetermined goal of abstinence (Collins et al., 2011, 2015, 2019, 2021), and thus engage a greater number of people (Haeny et al., 2021; Verissimo & Grella, 2017). Thus, future research would benefit from examining the role of PTSD on recovery goals other than abstinence during the period following residential SUD treatment, including reductions in substance-related harm and improvements in quality of life. Third, to inform targeted programming and services in residential SUD treatment, we assessed participants’ experiences of risk factors in residential SUD treatment. A limitation of this approach is that levels of these risk factors may have changed during the period of re-entry (e.g., participants may have experienced greater exposure to trauma reminders outside of residential SUD treatment, thereby increasing PTSD severity), and thus findings of the current study may have less utility for understanding treatment and service needs during the period of community re-entry. Investigations are needed to examine factors occurring during community re-entry that are proximally related to return to substance use, such as those that employ ecological momentary assessment (EMA) designs. Fourth, while participants did not receive empirically supported PTSD treatment in residential SUD treatment, we do not know whether they received these services during community re-entry; this would be an important question for future research. Fifth, we did not include biological corroboration of use or non-use (e.g., urine drug test) in the follow-up assessment, and it is possible that some participants may have provided inaccurate self-reports of their substance use. Despite these limitations, results of the current study highlight the importance of PTSD in return to substance use following residential SUD treatment. Our findings provide the impetus for future investigations of co-occurring PTSD and SUD during the high-risk period of immediate community reintegration following residential SUD treatment.

## Data Availability

The data that support the findings of this study are available from the corresponding author, Nicole H. Weiss, Ph.D., upon reasonable request.
